# Errors in medication history at hospital admission: prevalence and predicting factors

**DOI:** 10.1186/1472-6904-12-9

**Published:** 2012-04-03

**Authors:** Lina M Hellström, Åsa Bondesson, Peter Höglund, Tommy Eriksson

**Affiliations:** 1 eHealth Institute and School of Natural Sciences, Linnaeus University, Kalmar, Sweden; 2 Department of Medicines Management and Informatics, Skåne Regional council, Malmö, Sweden; 3 Department of Clinical Pharmacology, Lund University, Lund, Sweden

## Abstract

**Background:**

An accurate medication list at hospital admission is essential for the evaluation and further treatment of patients. The objective of this study was to describe the frequency, type and predictors of errors in medication history, and to evaluate the extent to which standard care corrects these errors.

**Methods:**

A descriptive study was carried out in two medical wards in a Swedish hospital using Lund Integrated Medicines Management (LIMM)-based medication reconciliation. A clinical pharmacist identified each patient's most accurate pre-admission medication list by conducting a medication reconciliation process shortly after admission. This list was then compared with the patient's medication list in the hospital medical records. Addition or withdrawal of a drug or changes to the dose or dosage form in the hospital medication list were considered medication discrepancies. Medication discrepancies for which no clinical reason could be identified (unintentional changes) were considered medication history errors.

**Results:**

The final study population comprised 670 of 818 eligible patients. At least one medication history error was identified by pharmacists conducting medication reconciliations for 313 of these patients (47%; 95% CI 43-51%). The most common medication error was an omitted drug, followed by a wrong dose. Multivariate logistic regression analysis showed that a higher number of drugs at admission (odds ratio [OR] per 1 drug increase = 1.10; 95% CI 1.06-1.14; p < 0.0001) and the patient living in their own home without any care services (OR = 1.58; 95% CI 1.02-2.45; p = 0.042) were predictors for medication history errors at admission. The results further indicated that standard care by non-pharmacist ward staff had partly corrected the errors in affected patients by four days after admission, but a considerable proportion of the errors made in the initial medication history at admission remained undetected by standard care (OR for medication errors detected by pharmacists' medication reconciliation carried out on days 4-11 compared to days 0-1 = 0.52; 95% CI 0.30-0.91; p=0.021).

**Conclusions:**

Clinical pharmacists conducting LIMM-based medication reconciliations have a high potential for correcting errors in medication history for all patients. In an older Swedish population, those prescribed many drugs seem to benefit most from admission medication reconciliation.

## Background

The problem of inaccurate medication lists at hospital admission and discharge is extensive [1-3] and has gained attention, specifically with regard to the issue of patient safety, in recent years [1,4]. An accurate medication list at hospital admission is essential for the evaluation and further treatment of patients, to prevent medication errors and adverse drug events in hospital and after discharge. Errors in the medication history are sometimes identified and corrected early enough to prevent any harm to the patient and are then of no clinical importance, although the administrative work can waste valuable time for the health care staff involved. Unidentified errors, however, can result in the patient receiving potentially harmful, inaccurate treatment. Possible causes for the errors in medication histories are multi-factorial, relating to the system, the patient, or the health care staff [1,5-7].

Medication reconciliation has been endorsed by patient safety organisations and authorities in a number of countries as a method of improving the accuracy of patients' medication lists [1,4,8]. The Institute for Healthcare Improvement in the United States has described medication reconciliation as being "the process of identifying the most accurate list of a patient's current medicines - including the name, dosage, frequency, and route - and comparing them to the current list in use, recognizing any discrepancies, and documenting any changes, thus resulting in a complete list of medications, accurately communicated" [1].

The Lund Integrated Medicines Management (LIMM) model offers a systematic approach for individualising and optimising drug treatment for inpatients [9]. The LIMM model has been continuously developed and implemented in a number of Swedish hospitals over more than ten years. This model includes a pharmacist intervention for medication reconciliation at admission, team interventions for medication reviews and monitoring during the hospital stay, and a discharge medication reconciliation procedure. Previous studies have associated the LIMM model with prescription of fewer inappropriate drugs [9,10] and reductions in the number of drug-related patient revisits to hospital [9,11] and primary care [11]. A smaller early study also suggested that using the LIMM medication reconciliation at admission would effectively identify errors in the medication history [12]. It is also important to carry out a more comprehensive evaluation of the subsequent actions of the pharmacists and medical practitioners. Evaluation of these actions (e.g. suggestions for change and changes made to the prescriptions) can provide insight into the factors responsible for the identified outcomes and suggestions for optimizing the intervention [13]. Furthermore, there is a need to determine whether it is possible to identify patients with the greatest risk of experiencing medication history errors at hospital admission. If those patients can be identified in clinical practice, it will enable better resource allocation, as interventions to prevent medication history errors can be directed towards the relevant groups. Results concerning which risk factors predict such errors in medication histories are currently contradictory [5-7,14-17].

The objective of this study was to describe the frequency and type of medication history errors identified by pharmacists performing medication reconciliations for patients admitted to a Swedish hospital, and to evaluate predictors for those medication errors. A secondary objective was to evaluate the degree to which standard care identifies errors in the medication history when the pharmacist's medication reconciliation is delayed.

## Methods

### Setting and population

This prospective study was conducted in two internal medicine wards (designated A and B) at the University hospital of Lund, Sweden. A LIMM-based clinical pharmacy service, including medication reconciliation at admission, was implemented in January and October, 2007, in the respective wards. All patients admitted to wards A and B after implementation of the service until the end of the year 2007, were eligible for inclusion in the study. Patients discharged or deceased before the pharmacist could conduct admission medication reconciliation were excluded from the study. There were 22 beds in each ward. The weekday staff in each ward comprised two junior physicians and two senior physicians, one clinical pharmacist, three nurses, three assistant nurses, one physiotherapist and one occupational therapist. Availability of beds alone decided the ward to which a patient was admitted. The wards used the standard hospital electronic health record (EHR) system (Melior^®^, Siemens Corp.); this was used in all hospital wards but was not used in primary care. The primary care centres in the region surrounding the hospital used either another EHR system or paper-based health records. Community care services used paper-based medication lists. The different levels of care (i.e. primary, secondary and community care) exchanged information about the patients' current medication lists by phone, fax, or mail. No electronic communications between the hospital and the primary care centres or community care services was possible at the time of the study. The regional ethical board of the University of Lund, Sweden, did not consider ethical approval to be necessary and had no objections to the study.

### Collection of data

A two-step procedure was used to collect and classify data on medication discrepancies and errors in the medication histories. Firstly, clinical pharmacists conducted medication reconciliations and documented their work in a LIMM medication interview questionnaire form (Additional file 1). Five different pharmacists worked at the wards during the study period. Secondly, two pharmacy students and a research pharmacist classified the identified discrepancies and errors.

### The medication reconciliation process in the LIMM model

The admission medication reconciliation process in the LIMM model is comprehensive and was developed over about 5 years and implemented on top of standard care [9,12]. Following a strict protocol, clinical pharmacists identified the patient's pre-admission medication list. For patients capable of participation and willing to participate, an initial medication interview was conducted. The pharmacist asked which medications and dosages the patient had been taking before admission. Specific questions were asked about the use of painkillers, heart medications, stomach medications, sleeping pills, anti-diabetics, eye drops, inhalation drugs, over-the-counter drugs and herbal drugs, in order to increase the probability of including all the patient's medications. Sometimes, a medical interview was conducted with a close relative instead of the patient. In addition (or otherwise, if an interview could not be conducted), the pharmacist consulted all available pre-admission lists, including drug lists from primary and community care, the national pharmacy register (all drugs dispensed within the past 15 months) [18], and prescription forms from the medication dispensing system ApoDos (a multi-dose system where all medications that the patient should be taking on one occasion are machine-packed together in small, fully labelled plastic bags at a pharmacy dispensing centre and delivered to the patient every second week) [19]. Based on this information, a list with the patient's prescribed medications was documented in the LIMM medication interview questionnaire, part 1 (Additional file 1). Parts 2 and 3 of the LIMM medication interview questionnaire were conducted with some patients; these parts comprised questions about knowledge of, practical handling of, adherence with, and beliefs about medications [9]. This paper reports the results from part 1 only.

The pre-admission medication list identified by the pharmacist was regarded as the most accurate list available since it was based on all available information sources and had been compiled according to a well established, systematic method [5,6,20-22]. Differences between this pharmacist-compiled pre-admission medication list and the medication list in the hospital EHR were documented in the LIMM medication interview questionnaire. The pharmacist consulted the patient's EHR to establish possible reasons for the differences. Discrepancies noted in the hospital medication list which the pharmacist judged relevant and possibly requiring correction were discussed with a ward physician. The pharmacist recommended corrections for the hospital EHR, and the physician then made the final decision and was responsible for correcting the hospital EHR list when necessary. The use of over-the-counter drugs and herbal drugs by the patients was also documented in the questionnaire and discussed with the physician when considered clinically relevant. The pharmacists' recommendations and the subsequent actions by the physician or pharmacist were documented in a medication review form.

It was ward policy that a clinical pharmacist should conduct the LIMM-based medication reconciliation within one day of admission to the ward or on Mondays for patients admitted on weekends. Medication reconciliation was conducted once for each patient and took on average 32 minutes per patient if a patient interview was conducted and 15 minutes if no interview was conducted [unpublished observations, personal communication Tommy Eriksson 27/03/2012]. This time included the face-to-face discussion with a physician about discrepancies in the hospital medication list.

Occasionally, it took longer than one day for the pharmacist to conduct the medication reconciliation. This was attributed to time constraints, lack of personnel, or temporarily closed wards because of an infection outbreak among the patients. If a clinical pharmacist was not available, physicians and/or nurses occasionally corrected errors in the medication history (standard care), but there were no instructions or forms for these changes, in contrast to the LIMM-based structured medication reconciliations.

### Definition and classification of medication discrepancies and errors

The identified differences between the pharmacist-acquired medication list and the medication list in the EHR were classified retrospectively by reviewing the LIMM medication interview questionnaires and the EHR. A medication discrepancy was defined as an addition or withdrawal of a drug, or a change to the dose or dosage form. An incorrect dosage interval was not defined as a discrepancy if the total dosage/24 h had not been changed. Changes to an equivalent generic drug or withdrawal of drugs with a long dosage interval, e.g. once monthly, were also not regarded as medication discrepancies. The medication discrepancies were further classified by type: drug omitted (the drug had not been registered in the hospital EHR drug list), additional drug (a drug had been erroneously added to the hospital EHR drug list), dosage too high, dosage too low, or wrong dosage form.

Medication discrepancies for which the reviewing pharmacists could not identify any clinical reason (unintentional changes) were deemed to be medication errors. Our definition of a medication error was based on the definition proposed by Leape [23]: "A medication error is any error in the process of prescribing, dispensing or administering a drug, whether there are adverse consequences or not". There were two exceptions to this: that only errors in the medication history were included, and that discrepancies corrected before reaching the patient were not considered medication errors. For example, discrepancies concerning weekly doses that were identified before the dosing occasion or involving omission of drugs that were to be given as needed and which the patient had not yet required were not counted as errors. Over-the-counter drugs and herbal drugs were not included in the drug list in the EHR and hence could not result in medication discrepancies or errors.

Two pharmacy students (doing their Masters theses) were responsible for the classification of medication discrepancies and errors as described above. They were informed by a research pharmacist (ÅB) about the classification procedure and they continually discussed any lack of clarity with the research pharmacist. To evaluate the percent of cases which the raters agreed upon, 30 patients were classified independently by the two students and one research pharmacist (LH). The agreement with regard to the number and type of medication errors was 83%.

### Statistics

An open source software based on the R language and environment for statistical computing (http://www.R-project.org) was used for all statistical analyses [24]. Descriptive statistics are shown as medians (interquartile range), means (95% confidence intervals, CI) and frequencies or percentages (95% CI) when appropriate. The denominator for calculating the error rate was the number of prescribed medications in the hospital EHR before medication reconciliation, plus any medications omitted. A multivariable binary logistic regression analysis was conducted where the response variable was the presence or absence of medication errors. The following model variables were pre-specified potential predictors: number of drugs at admission (every increase by one drug), age (every 10 years' increase), sex (0 = Female, 1 = Male), type of care service before admission (0 = Living in own home with no care service, 1 = Living in own home but enrolled in community home care services, 2 = Living in care home), and directly admitted to the study ward without transferral from another ward (0 = No, 1 = Yes). In addition, the extent to which standard care identified medication errors was evaluated by including the number of days from admission to the ward until medication reconciliation (0 = 0-1 days, 1 = 2-3 days, 2 = 4 days or more) in the model. This variable was not considered a potential predictor but was included in order to evaluate if the medication errors remained undetected by standard care if the LIMM admission medication reconciliation was delayed. The variables "2-3 days" and "4 days or more" were not targeted controls; the medication reconciliation was delayed because of time constraints or other factors and never deliberately. No variables were eliminated from the regression model. Eleven percent of the data for the variable "directly admitted to the study ward", and 10% for "number of days from admission to the ward until medication reconciliation" were missing. Data were complete for the other variables. In the multivariable regression model, missing data was imputed using a multiple imputation method [25]. The le Cessie-van Houwelingen-Copas-Hosmer unweighted sum of squares test for global goodness of fit was used to assess the fit of the model [25]. The significance level in the analysis was set to 0.05.

## Results

### Description of the study sample

In total, 818 patients were eligible for inclusion. Patients who did not receive a medication reconciliation (n = 137) and patients for whom important demographic and study data were missing (n = 11) were excluded. The final study population comprised 670 patients: 524 in ward A and 146 in ward B. The characteristics of the study population are summarised in Table 1. Most of the patients (62%) were aged over 80 years and 66% had been prescribed more than five drugs for regular use before admission to hospital. Seven percent were younger than 65 years. There were no differences in patient characteristics between wards A and B.

**Table 1:** Characteristics of the study participants (n = 670)

Age, mean, in years (SD)	81 (10)
Sex, % female	53%

Number of drugs at admission, median (IQR)	
Regular use	7 (5-11)
As needed	1 (0-2)

Patients using a multi-dose system (ApoDos^®^), % of patients	21%

Length of stay in the ward, median days (IQR)	8 (4-12)

Number of drugs at discharge, median (IQR)	
Regular use	8 (5-11)
As needed	1 (1-3)

SD, standard deviation. IQR, interquartile range

### Description of the medication reconciliation process

**Figure 1:**
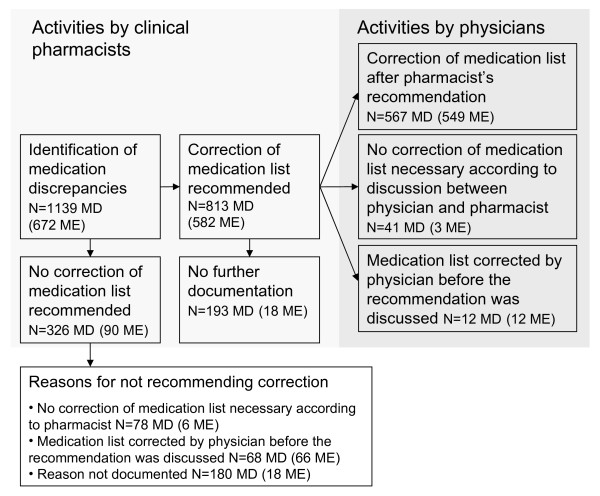
**Illustration of the medication reconciliation process**. The figure includes the number of identified medication discrepancies (MD), the number of pharmacist-suggested changes, and the number of corrected drug prescriptions. The number of discrepancies that were later classified as medication errors (ME) by the reviewing pharmacists is also given.

The clinical pharmacists identified 1136 medication discrepancies between the lists for 420 of 670 patients (63%; 95% CI 59 to 66%). The mean of medication discrepancies in the total cohort was 1.7 (95% CI 1.6-1.8) per patient, and the mean per affected patient was 2.7 (95% CI 2.5-2.9). The actions of the pharmacists and physicians after the initial identification of discrepancies are summarised in Figure 1. The pharmacists recommended correction of 813 discrepancies (71%). For 567 of the 813 suggestions (70%), the medication list was corrected accordingly by the physician. In 193 cases (24%), it was unknown whether the medication list was corrected after the pharmacist's recommendation. Either the discrepancy was no longer relevant for the patient, or the pharmacist did not document the results because of lack of time, or the physician never decided on an eventual correction.

### Frequency and types of errors in medication history

Of the 1136 identified medication discrepancies, 672 (59%) were classified as medication errors. These errors affected 313 patients, representing a frequency of 47% (95% CI 43 to 51%). The error rate was 10.2% (672/6582) of the total number of prescribed drugs at hospital admission. The overall mean (in the total cohort) was 1.0 (95% CI 0.9-1.1) medication error per patient, and the mean per affected patient was 2.1 (95% CI 1.9-2.3). Twenty-three percent of the patients had one error, 11% had two errors and 13% had between three to nine errors. The most common medication error was omission of a drug, followed by a wrong dose. The frequencies of the various types of medication error and the most commonly associated drug classes are shown in Table 2. Overall, 93% (n = 627) of the medication errors resulted in correction of the EHR medication lists, as a result of either the pharmacist's suggestion (n = 549) or the physician's initiative before the pharmacist's suggestion (n = 78) (Figure 1).

**Table 2:** Number of medication errors by type, and most frequent ATC codes for each type of error.

Type of error	Medication errors; numbers (%)	The three most frequent ATC codes by type of error; number of errors					
		**B03**	**C07**	**N02**	**N05**	**N06**	**R03**
		
Omission of drug	417 (62)			37	37		47
Dose too high	86 (13)	8		9	11		
Dose too low	82 (12)			11	12	7	
Additional drug	79 (12)			7	8		8
Wrong dosage form	8 (1)		3	3			2
**Total**	**672 (100)**			**67**	**68**		**57**

ATC-codes: B03, Antianemic preparations; C07, Beta-blocking agents; N02, Analgesics; N05, Psycholeptics; N06, Psychoanaleptics; R03, Drugs for obstructive pulmonary disease

ATC, Anatomic Therapeutic Chemical

### Predicting errors in medication history

In the multivariable logistic regression model, an increased number of drugs at admission and absence of any care service before hospital admission (i.e. patients living in their own homes without community care service) were significant predictors for medication errors (Table 3). The odds for a patient experiencing at least one medication error increased by 10% for every additional medication at admission (OR 1.10; 95% CI 1.06-1.14). Among patients living in their own homes without any care service, 48% experienced at least one error compared to 47% of those living in a care home (OR 1.58; 95% CI 1.02-2.45). The remaining prediction factors included in the regression model showed no correlation with the risk of experiencing medication errors.

**Table 3:** Predictors of errors in the medication history at admission to hospital.

Potential predictors		Number of patients with an error (%)	Odds ratio (95% CI)
Number of drugs at admission	For each 1-drug increase	313 (47)	1.10 (1.06-1.14)*

Age	For each 10-yr increase	313 (47)	1.08 (0.92-1.27)

Ward	A	250 (48)	Reference
	
	B	63 (43)	0.82 (0.56-1.21)

Sex	Male	135 (43)	Reference
	
	Female	178 (50)	1.33 (0.96-1.83)

Type of care service before admission	Care home	63 (47)	Reference
	
	Own home with community care services	74 (45)	1.08 (0.67-1.75)
	
	Own home, no care service	176 (48)	1.58 (1.02-2.45)*

Directly admitted to study ward **^a^**	Yes	155 (47)	Reference
	
	No; transferred from another ward	131 (49)	1.12 (0.80-1.57)

**Days until medication reconciliation**			

Number of days until the pharmacist's medication reconciliation **^a^**	0-1 days	168 (51)	Reference
	
	2-3 days	101 (49)	0.85 (0.59-1.23)
	
	4-11 days	24 (38)	0.52 (0.30-0.91)*

Odds ratios were derived from a multivariable binary logistic regression model

* p < 0.05

^a^Number (%) of patients is reported only for those with complete data. In the logistic regression model missing data was imputed

### Correction of errors in medication history by standard care

As the variable for the number of days until medication reconciliation was included in the regression model, it can be assumed that the patients reconciled on days 4-11 had the same adjusted rate of medication error when they were admitted to hospital as the rest of the patients. The odds ratio for medication errors detected by pharmacist medication reconciliation was 0.52 (95% CI 0.30-0.91) on days 4-11 (Table 3). The results thus suggest that, in a statistically significant proportion of the patients reconciled on days 4-11 who had medication errors at admission, the errors had already been corrected at the time of pharmacist medication reconciliation and that standard care was responsible for these corrections. In the remaining patients who had medication errors in their EHRs at admission, the errors were still undetected by standard care after 4-11 days and instead identified by the pharmacist medication reconciliation. Within 2-3 days after admission, the probability that a pharmacist would detect the errors was as high as that on days 0-1 (Table 3), i.e. standard care did not correct significantly more errors when the pharmacist's medication reconciliation was moderately delayed.

## Discussion

In our study population of mainly older patients, approximately 50% were affected by errors in the medication history at admission to hospital. The most common error was the erroneous omission of a drug from the hospital EHR medication list. Predictors of the occurrence of medication errors included an increased number of preadmission drugs and living in one's own home without community care service.

Our findings are similar to those of other studies [2,17,20], but there are also reports of lower [15] or higher [2,6] rates of error in medication histories at hospital admission. Different definitions of medication discrepancies and errors and variability in methods of data collection could explain the differences between studies and make it difficult to compare rates of error across studies.

The association between the number of prescribed drugs at admission and the occurrence of medication errors was not surprising. Previously, some researchers have found associations between errors in the medication history and the number of drugs at admission [5,14], but some have not [6,15,16]. To the best of our knowledge, the association between absence of any care service before admission and medication errors has not been previously suggested. It is likely that the type of care service is not, in itself, important. Rather, the availability of a current medication list at hospital admission might be the important underlying factor. Patients in community care or care homes often take a current medication list with them to hospital, possibly facilitating the recording of the initial medication history by a physician or nurse and subsequently lowering the risk for medication errors. However, the absolute difference between the groups (48% vs 47% of patients with an error, as seen in Table 3) was small and the value of this predictor in clinical practice would be limited. Also, the influence of this predictor is likely to vary substantially between settings and above all between countries; it will depend on the level of communication between community care services and the hospital, and on the routines for the patient taking their medication lists or medications with them when attending the emergency department.

There are varying results from other research on predictors for errors in the medication history. In accordance with our results, Gleason and colleagues found that there were few predictors associated with medication errors at admission and they suggested that well-designed processes for medication history verification were more important than patient characteristics [5]. In contrast to our results, some researchers have found that higher age [5,14,15] is a significant predictor. However, the patients in our study wards were older than those in previous studies [5,14,15], and our results might have differed if the patient cohort had been younger. Previous studies have identified significant predictors for errors in the medication history which were not included in our regression model, e.g. certain "high-risk" drugs, many outpatient visits during the previous year, and staffing levels [6,7].

The relative importance of the medication reconciliation by a pharmacist in terms of added value compared to standard care was also of interest. Optimally, this should be studied in a randomized, controlled trial. Because we were unable to carry out a randomized trial, we used an indirect measure to evaluate the degree to which standard care corrected medication errors. The pharmacists did not conduct the medication reconciliation until 4-11 days after admission for 20% of the patients due to time constraints or lack of personnel. If standard care had not identified and corrected any medication errors at all, the probability that the pharmacists identified medication errors on days 4-11 would have been as high as that on days 0-1. However, regression analysis suggested that the probability that there would still be an error in a patient's medication history after 4 or more days in the ward was lower compared to days 0-1. This implies that standard care had had partly corrected the errors in affected patients by that time, but a considerable proportion of the errors made in the initial EHR medication history at admission remained undetected by standard care. Two to three days after admission, the probability that patients would still be prescribed the wrong drug or dose was as high as the first day after admission. The potentially severe nature of some of the errors in medication history [5,17,21] underlines the importance of reconciling the medication list soon after admission to avoid patient harm as a consequence of error, preferably within 24 hours of admission.

We believe it is necessary for medication reconciliation processes to be well designed and systematic, and aided by structured forms and detailed guidelines. Clinical pharmacists, as key members of a multidisciplinary team, are very well suited to perform such systematic medication reconciliations. A review by the British National Institute for Health and Clinical Excellence [1] showed that there is evidence that pharmacist interventions are the most effective among the studied medication reconciliation interventions. However, it was commented that the current evidence is poor and further comparative studies of different medication reconciliation programs will be needed to reveal which approach is most effective from a clinical and economic perspective. There are many promising and emerging technologies that may be effective in medicines reconciliation as well. Nonetheless, our results highlight the benefits of structured reconciliations by pharmacists over occasional reconciliations as part of standard care.

The physicians' acceptance of the pharmacists' recommended changes to drug therapy is often used in studies of clinical pharmacy services as a measure of quality. In this study, 94% of the recommendations from the pharmacist concerning errors in medication history were accepted and implemented by the physicians, which suggests that the process was effective. In a number of cases, there was no information about the measures taken by the physicians after the pharmacists' recommendations, or about the pharmacists' reasons for not recommending changes to the medication list. More precise documentation might have provided even better insight into the effectiveness of the process.

This study adds to the evidence that LIMM-based patient care in hospital offers a positive contribution. We detected and corrected medication errors in almost half of the study patients. Although this study did not evaluate possible harm from these errors, a study including a sample of our study patients reported the clinical significance of pharmacists' recommendations [26]. Recommendations were ranked by two physicians on a six-point scale from 1 (adverse significance) to 6 (extremely significant). Of 70 recommendations, 59% were ranked somewhat significant, 23% significant and 10% very significant. Seven percent had no significance and one recommendation was judged to have adverse significance. However, this case did not result in documented patient harm. Fifty-six of our study patients were also followed up as part of an intervention study [9]. That study showed that LIMM-based medication reconciliation at admission and medication reviews in hospital improve the appropriateness of drug therapy and may also decrease drug-related revisits to hospital. The results of the admission process (i.e. the correction of medication errors) in the present study are therefore very likely to be at least partly responsible for these positive clinical outcomes [9].

This study had several limitations. Firstly, it was conducted in an internal medicine population in a single hospital, which limits the generalisability. Secondly, acceptance of the pharmacist-acquired medication list as the most accurate preadmission drug list available could be questioned. It is possible that some medication discrepancies escaped our detection. However, studies have shown that pharmacists appear to be especially suited and more effective than physicians when obtaining medication histories [22] and the methods used by pharmacists to obtain medication histories are well established [5,6,20,21]. Our method was strengthened by the fact that the pharmacists used a number of different information sources apart from the patient interview; for example, pharmacy records are known to improve the accuracy of medication lists [27]. The pharmacists were also well informed of the requirements and followed a strict protocol for the medication reconciliation process. Thirdly, the classification of discrepancies into medication errors partly relies on subjective judgment and is therefore subject to bias.

## Conclusions

We conclude that medication history errors at hospital admission are common, which highlights the importance of introducing processes for ensuring that the medication lists are accurate and complete as soon as possible after admission. Clinical pharmacists can be valuable in performing structured medication reconciliations to reduce the risk of medication errors. Our findings suggest that there is limited potential for predicting which patients are at highest risk of experiencing errors in their medication history. More research is needed, particularly to uncover the reasons for the possible impact of pre-admission care services on medication errors. In general, we believe that systematic medication reconciliations should be conducted in all patients admitted to hospital. Among older patients admitted to Swedish hospitals, those being prescribed many drugs could benefit the most from admission medication reconciliations by clinical pharmacists.

## Competing interests

The authors declare that they have no competing interests.

## Authors' contributions

All authors were involved in designing the study. ÅB, TE and LH were involved in the collection of data. ÅB, TE, PH and LH interpreted the data. LH and PH were responsible for the statistical analysis. LH drafted the first version of the manuscript and all authors made critical revisions to the manuscript. All authors gave final approval to the manuscript.

## Pre-publication history

The pre-publication history for this paper can be accessed here:

http://www.biomedcentral.com/1472-6904/12/9/prepub

## Supplementary Material

Additional file 1 **LIMM Medication Interview Questionnaire**. The LIMM Medication Interview Questionnaire used by the clinical pharmacists when conducting admission medication reconciliation. Click here for file
