# Supplementary material for: Errors in medication history at hospital admission: prevalence and predicting factors
Source: BMC Clin Pharmacol. 2012 Apr 3;12:9. doi: 10.1186/1472-6904-12-9 (PMC3353244; doi:10.1186/1472-6904-12-9)
Supplement: Additional file 1 — LIMM Medication Interview Questionnaire. The LIMM Medication Interview Questionnaire used by the clinical pharmacists when conducting admission medication reconciliation. [file 1472-6904-12-9-S1.PDF]

LIMM Medication Interview Questionnaire

**Ward**	**Bed**	**Name**	**Date of birth**	**Date and signature**	**Follow up,** date, sign
**Do you handle your medications yourself?** ○ No ○ Yes			**Apodos?*** ○ No ○ Yes, version:		

Part 1: Medication reconciliation

Part 2: If handling medications oneself

**Medications in hospital prescription order**					**Pre-admission medications** **Dosing §**			Present problem (x) No problem (✓)		
Date started	**Medication name, dosage form, strength**	**Dosing**	**Comments**	Date stopped			**Suggested correct list**	Indi-cation	Adher-ence	**Follow up**

* Apodos®: multi-dose system with machine-packed medicines
§ Indicate which information sources used: patient/kindred (PA), primary care (PC), community care (C), Apodos, pharmacy register (PR). Please document the latest dispensing date in the pharmacy register for each drug.

Part 1: Are you using any other medications? ☐ eyedrops ☐ inhalers ☐ painkillers ☐ heart medications ☐ stomach medications ☐ sleeping pills ☐ antidiabetics ☐ OTC drugs ☐ herbal drugs ☐ drugs as per needed. How often do you take these?
Part 2: Practical handling problems? ☐ Swallowing, crushing/splitting ☐ Opening bottles or blisters ☐ Inhaling
Adverse drug reactions?

☐ Patient consent for using pharmacy register:
Date **Signature**

**Other information from the interview**	
**Number of discrepancies in medication list:**	
